# Chicken ovalbumin upstream promoter transcription factor II regulates uncoupling protein 3 gene transcription in *Phodopus sungorus*

**DOI:** 10.1186/1471-2199-8-1

**Published:** 2007-01-04

**Authors:** Tobias Fromme, Kathrin Reichwald, Matthias Platzer, Xing-Sheng Li, Martin Klingenspor

**Affiliations:** 1 Department of Animal Physiology, Faculty of Biology, Philipps-University, D-35043 Marburg, Germany; 2 Genome Analysis, Leibniz-Institute for Age Research – Fritz Lipmann Institute, D-07745 Jena, Germany

## Abstract

**Background:**

Ucp3 is an integral protein of the inner mitochondrial membrane with a role in lipid metabolism preventing deleterious effects of fatty acids in states of high lipid oxidation. *Ucp3 *is expressed in brown adipose tissue and skeletal muscle and controlled by a transcription factor complex including PPARalpha, MyoD and the histone acetyltransferase p300. Several studies have demonstrated interaction of these factors with chicken ovalbumin upstream promoter transcription factor II (Coup-TFII). This nuclear receptor is involved in organogenesis and other developmental processes including skeletal muscle development, but also co-regulates a number of metabolic genes. In this study we *in silico *analyzed the upstream region of *Ucp3 *of the Djungarian hamster *Phodopus sungorus *and identified several putative response elements for Coup-TFII. We therefore investigated whether Coup-TFII is a further player in the transcriptional control of the *Ucp3 *gene in rodents.

**Results:**

By quantitative PCR we demonstrated a positive correlation of *Coup-TFII *and *Ucp3 *mRNA expression in skeletal muscle and brown adipose tissue in response to food deprivation and cold exposure, respectively. In reporter gene assays Coup-TFII enhanced transactivation of the *Ucp3 *promoter conveyed by MyoD, PPARalpha, RXRalpha and/or p300. Using deletions and mutated constructs, we identified a Coup-TFII enhancer element 816–840 bp upstream of the transcriptional start site. Binding of Coup-TFII to this upstream enhancer was confirmed in electrophoretic mobility shift and supershift assays.

**Conclusion:**

Transcriptional regulation of the *Coup-TFII *gene in response to starvation and cold exposure seems to be the regulatory mechanism of *Ucp3 *mRNA expression in brown adipose and skeletal muscle tissue determining the final appropriate rate of transcript synthesis. These findings add a crucial component to the complex transcriptional machinery controlling expression of *Ucp3*. Given the substantial evidence for a function of Ucp3 in lipid metabolism, Coup-TFII may not only be a negative regulator of glucose responsive genes but also transactivate genes involved in lipid metabolism.

## Background

Uncoupling protein 3 (Ucp3) is a member of the family of uncoupling proteins, which are located in the inner mitochondrial membrane and uncouple the respiratory chain from ATP synthesis by dissipating the proton motive force [1,2]. The physiological function of Ucp3 is subject to an ongoing debate [3]. Regulation of *Ucp3 *expression suggests a role in lipid metabolism. Skeletal muscle *Ucp3 *transcription is increased in response to food deprivation, a robust mechanism consistently observable in man, rodents and even fish [4]. Further physiological conditions positively regulating *Ucp3 *include cold exposure [5,6], acute exercise [7] and streptozotocin-induced diabetes [8]. Increased levels of circulating free fatty acids (FFA) are common to all these physiological states; infusion experiments imply that these are the primary cause for *Ucp3 *upregulation [9]. Therefore it has been suggested, though not proven experimentally, that Ucp3 is a fatty acid anion carrier [10].

The biochemical properties of the protein as measured in mitochondrial proton leak assays by parallel recording of membrane potential and oxygen consumption infer a role for Ucp3 in the defense against radical oxygen species (ROS), mitigating their generation by mild uncoupling [11]. This possible function is corroborated by the finding that a product of ROS induced lipid peroxidation, 4-hydroxy-2-nonenal, specifically induces uncoupling by Ucp3 and that even a small reduction of membrane potential markedly decreases ROS production [12]. A controversial hypothesis which takes into account both physiological and biochemical data emphasizes that the export of fatty acids or hydroperoxy fatty acids and subsequent protonated re-influx of a certain fraction into the matrix would result in a net proton import detectable as mild uncoupling [13,14]. This mechanism would reduce ROS production and at the same time reduce the level of non-esterified fatty acids in the matrix susceptible to peroxidation, thereby preventing deleterious effects in states of high lipid oxidation.

*Ucp3 *is predominantly expressed in skeletal muscle (SKM) and brown adipose tissue (BAT), both tissues with exceptionally high lipid oxidation capacities. The principal molecular constituents of *Ucp3 *gene regulation have recently been identified by several copious studies. Heterodimers of peroxisome proliferator activated receptor α (PPARα) and the retinoic X receptor α (RXRα) bind to a response element (PPRE) within the proximal promoter region and activate transcription depending on the presence of myogenic differentiation antigen 1 (MyoD). MyoD binds to a series of non-canonical E-boxes directly adjacent to the transcriptional start site (TSS). Induction is enhanced by the coactivator p300 protein (p300), which acetylates MyoD and possibly surrounding histones [15]. Furthermore, the PPRE in the *Ucp3 *promoter is multifunctional, i.e. can alternatively be targeted by heterodimers of the thyroid hormone receptor (TR) and RXRα stimulating expression in the presence of MyoD [16]. The ligands of PPARα (fatty acids) and TRs (T3) along with the requirement of MyoD indicate that this mechanism is involved in the acute response of *Ucp3 *expression in SKM to physiological stimuli. However, to our knowledge neither BAT-specific nor differentiation specific regulation has been characterized in detail to date.

Notably several studies have demonstrated interaction of PPARs, MyoD and/or p300 with chicken ovalbumin upstream promoter transcription factor II (Coup-TFII, official gene name: *Nr2f2*) [17-20]. This nuclear receptor is involved in organogenesis and other developmental processes [21] including SKM development [22], but also co-regulates a number of metabolic genes [18,23-25].

In this study we analyzed the upstream region of *Ucp3 *of the Djungarian hamster *Phodopus sungorus in silico *and identified several putative response elements for Coup-TFII. We therefore investigated whether Coup-TFII is a further player in the transcriptional control of the *Ucp3 *gene in rodents.

## Results

### Structure of the hamster Ucp3 gene

We successfully cloned the genomic *Ucp3 *locus of the hamster. Primers to amplify fragments of the upstream region (approx. -3500 to +50) were deduced from conserved segments of corresponding rodent genomic sequences. Resulting PCR products were sequenced and served as a framework to select primers for gap closure. We extended this contig to the putative 5' adjacent gene. *Ucp3 *introns were amplified with exonic primers based on the known hamster cDNA [GenBank: AF271265] and on the exon 1 sequence obtained as described above. The resulting genomic contig of 12,720bp included 3632 bp of *Ucp3 *5' gene flanking region as well as all *Ucp3 *exons and introns; the sequence of the terminal exon extends 210 bp 3' of the stop codon [GenBank: AY523564].

We identified two novel splice variants of the *Ucp3 *transcript in BAT [GenBank: DQ244043, DQ244044; not shown]. One transcript is characterized by partial mobilization of the second intron, presumably resulting in a premature stop [GenBank: DQ244044], the other lacks the second exon, possibly leading to alternative usage of the next in frame start codon wich is located in exon3 [GenBank: DQ244043].

**Figure 1:**
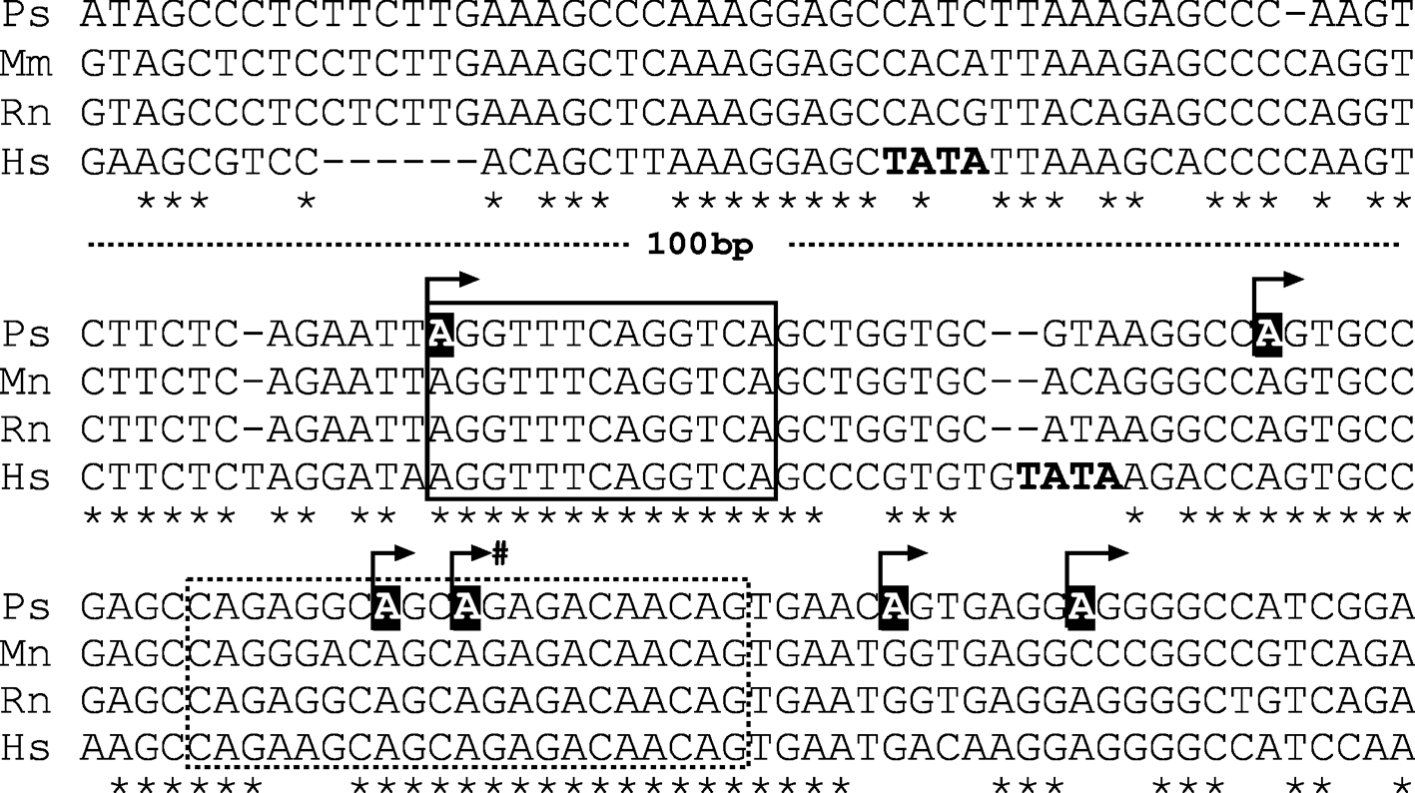
**Alignment of the human and three rodent proximal *Ucp3 *promoter regions**. Positions of identical bases are marked (*). The PPRE/TRE (box) as well as the MyoD binding triple E-box (dashed box) are highly conserved. Two TATA-like boxes (**bold**) can only be found in the human promoter. Transcriptional start sites of *P. sungorus *were identified within a wide range (black background), the arbitrarily chosen reference TSS is indicated (#). Rn = *R. norvegicus*, Mm = *M. musculus*, Ps = *P. sungorus*, Hs = *H. sapiens*

In our 5' RACE analysis, the TSS was variable within a range of 72 bp (Fig. 1). To determine a reference point for relative sequence positions we arbitrarily chose position 3632 of [GenBank: AY523564], represented by two clones obtained in BAT and SKM cDNA RACE experiments, respectively. Of six additional sequenced clones, one contained a start site at -49 (SKM), two at -20 (both BAT), one at -3 (SKM), one at +16 (SKM) and at +23 (BAT). Thus the TSS did not show a distinct tissue specificity.

### Analysis of the UCP3 promoter

We compared rodent and human proximal promoter sequences to classify promoter elements by their conservation (Fig. 1). Of the elements recently characterized by Solanes and coworkers (2003, 2004) – mediating transcriptional activation by PPARα, TRs and MyoD – the PPRE/TRE was located within a region that is identical in the compared species. The 21 bp long MyoD-binding triple E-box only differs in three nucleotides of the first repeat and is otherwise identical. In contrast, two TATA-like boxes identified in the human promoter [26] are absent in the known rodent sequences. By *in silico *analysis of the hamster *Ucp3 *promoter we detected several putative nuclear receptor binding sites, among them 28 elements predicted to possibly bind Coup-TFII.

### Sequencing of hamster Coup-TFII and qPCR

**Figure 2:**
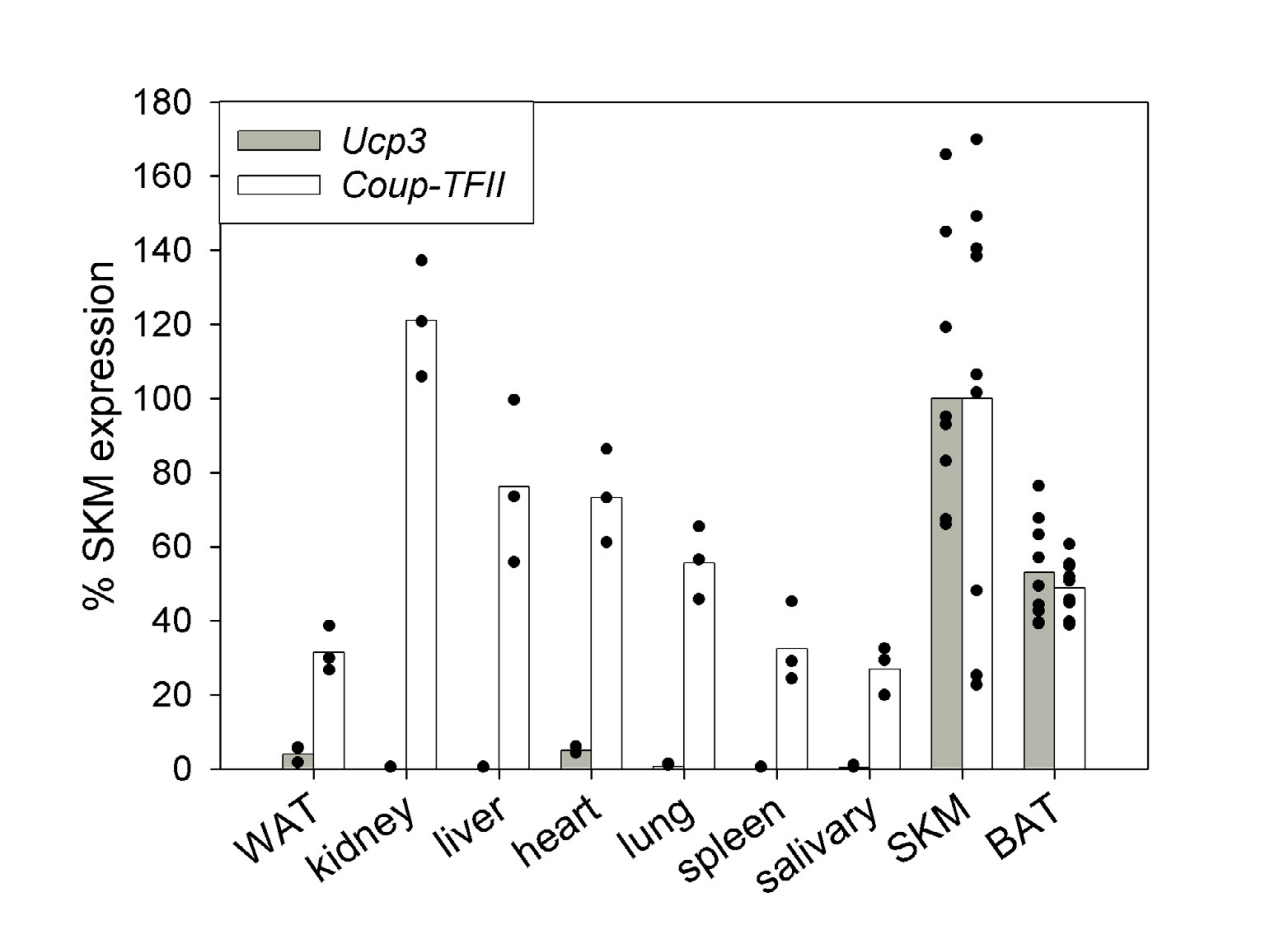
**Expression level of *Coup-TFII *and *Ucp3 *mRNA in tissues of *P. sungorus***. *Coup-TFII *mRNA is expressed in all examined tissue types as measured by qPCR. *Ucp3 *mRNA is found together with *Coup-TFII *mRNA in BAT and SKM. Both display highest variability in SKM (SKM/BAT n = 9, other tissues n = 3). Black dots represent individual results, bars are mean values. WAT = white adipose tissue, SKM = skeletal muscle, BAT = brown adipose tissue.

To derive homologous primers for qPCR we cloned and sequenced a 325 bp fragment of *Coup-TFII *[GenBank: DQ44042] comprising coding regions of exon 2 and 3. Hamster sequences were 98% identical with the murine orthologue. The deduced amino acid sequences of hamster and mouse are identical. In a first experiment we determined mRNA expression levels of *Coup-TFII *and *Ucp3 *by qPCR analysis in a panel of nine tissues of hamsters housed under standard laboratory conditions and fed *ad libitum *(Fig. 2). *Ucp3 *levels were highest in SKM and BAT, much lower in heart and white adipose tissue and virtually absent in all other tested tissues. *Coup-TFII *was well detectable in all analyzed tissues with highest amounts in kidney and SKM. The individual variation for both *Ucp3 *and *Coup-TFII *was maximal in SKM. *Ucp3 *mRNA levels in BAT were about 50% of SKM expression, interestingly the same is true for *Coup-TFII*.

**Figure 3:**
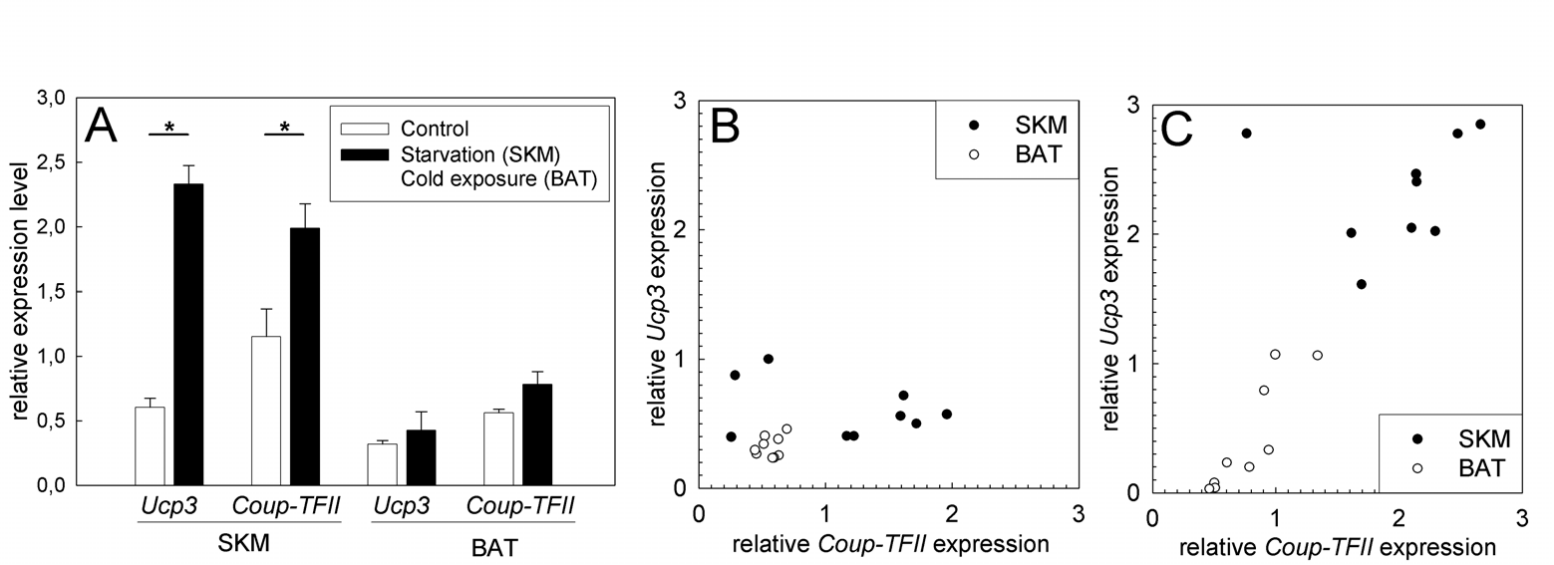
**Response of *Coup-TFII *and *Ucp3 *mRNA expression to physiological challenges inducing lipid utilization**. (**A**) Expression of *Coup-TFII *and *Ucp3 *mRNA in BAT of cold exposed and SKM of food deprived hamsters as compared to control conditions. Shown are mean values with standard error, * = p < 0,05. (**B**) Relationship of *Ucp3 *and *Coup-TFII *mRNA levels in SKM and BAT of hamsters housed under standard conditions with *ad libitum *access to food; r^2 ^= 0.211, p = not significant (SKM n = 9, BAT n = 9). (**C**)Correlation of *Ucp3 *and *Coup-TFII *mRNA abundancies in SKM of food deprived and BAT of cold exposed animals; r^2 ^= 0.834, p < 0.001 (SKM n = 9, BAT n = 9).

To explore a possible coregulation of *Ucp3 *and *Coup-TFII *we exposed hamsters to cold and food deprivation, physiological challenges known to upregulate *Ucp3 *expression [6,27] (Fig. 3A). In SKM food deprivation led to a significant induction of *Ucp3 *and *Coup-TFII*. In BAT of cold exposed hamsters *Ucp3 *was not systematically upregulated but displayed an increased individual variation. A similar degree of variation without significant cold-induced regulation was observed for *Coup-TFII*. To test for a possible coregulation we measured *Ucp3 *and *Coup-TFII *transcript abundancies and compared respective levels of control and challenged animals (Fig. 3B and 3C). Under control conditions no correlation was observed. However, we detected a highly significant correlation (r^2 ^= 0.834, p < 0.001) between *Ucp3 *and *Coup-TFII *levels in challenged animals.

### Luciferase reporter gene assays with cotransfected transcription factors

**Figure 4:**
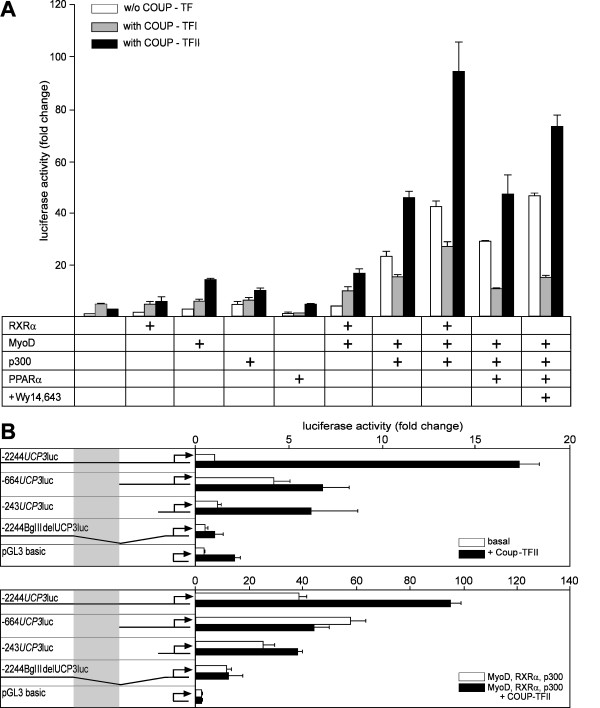
**Coup-TFII transactivates the *Ucp3 *promoter in cotransfection reporter gene assays**. (**A**) Activity of reporter gene construct -2244*UCP3*luc in cotransfection experiments with various transcription factors (fold change relative to basal -2244*UCP3*luc level). Coup-TFII coactivates the *Ucp3 *promoter in all combinations. It is especially effective together with MyoD, RXRα and p300. Coup-TFI does not activate reporter gene expression demonstrating a specific effect of Coup-TFII. (**B**) Identification of the region mediating Coup-TFII transctivation by analysis of deletion reporter gene constructs. Shown in the upper panel is the basal activity and induction by Coup-TFII of all constructs, in the lower panel the experiment was repeated on a background of MyoD, RXRα and p300 (note the different axis scaling). The shaded area highlights the region that is exclusively found on -2244*UCP3*luc and is responsible for the Coup-TFII effect in both setups.

We tested Coup-TFII in cotransfection reporter gene assays with regard to its potential in coactivating a *Ucp3 *promoter fragment of 2244 bp cloned into the luciferase reporter gene vector pGL3basic (-2244*Ucp3*luc). To evaluate the influence of Coup-TFII we compared cotransfections with or without expression vectors for PPARα, MyoD, RXRα and/or the histone acetyltransferase p300, factors known to regulate *Ucp3 *expression [15] (Fig. 4A).

Without Coup-TFII none of the tested factors upregulated -2244*Ucp3*luc more than 5-fold. Cotransfection of several factors exhibited a synergistic effect, which was augmented by p300, e.g. adding RXRα to MyoD hardly increased reporter gene activity, but in the presence of p300 notably induced expression. The strongest induction (47-fold) was achieved by a cotransfection of PPARα, MyoD and p300 in the presence of the synthetic PPARα agonist Wy14,643.

Cotransfection of Coup-TFII systematically increased reporter gene expression in all combinations tested. Together with a single other factor it was particularly effective with MyoD or p300, attaining an additional 3–5 fold induction. In combination with multiple factors Coup-TFII cotransfection consistently led to an approximate doubling of induction. The maximal induction in comparison to the basal construct activity was achieved in combination with RXRα, MyoD and p300 (95-fold), which is two fold higher than the maximal level observed in any experiment without Coup-TFII (ligand-activated PPARα + MyoD + p300, 47-fold). The dimension of induction by Coup-TFII is illustrated by comparison with the PPARα activation through the ligand Wy14,643 on a background of PPARα, MyoD and p300. Here transactivation by Coup-TFII and the ligand Wy14,643, respectively, is in the same order of magnitude.

To exclude that overexpression of any nuclear receptor or Coup-TF family member unspecifically leads to an induction of the *Ucp3 *promoter we utilized an expression vector for Coup-TFI, which is a close Coup-TFII relative sharing 85.5% of identical amino acid sequence, as a control. Replacing Coup-TFII by Coup-TFI in cotransfections with transcription factor combinations did not lead to activation of the *Ucp3 *promoter, but conversely downregulated reporter gene activity (Fig. 4A), possibly due to competition for RXRα. We therefore conclude that Coup-TFII specifically transactivated the *Ucp3 *promoter in synergy with other known transcription factors and coactivators.

To locate the responsible *cis *element, we studied the potential of Coup-TFII to activate several reporter gene deletion constructs (Fig. 4B). Coup-TFII transfection alone in this assay increased luciferase activity on -2244*Ucp3*luc 16-fold while all other constructs are much less sensitive. There is, however, a certain unspecific effect on the empty pGL3 basic vector, that can also be seen for all constructs used. This handicap was absent when we cotransfected the deletion constructs with MyoD, RXRα and p300, the combination exhibiting the strongest effect on -2244*Ucp3*luc. The Coup-TFII effect is limited to -2244*Ucp3*luc, locating the crucial region between nucleotides -1307 to -664, which are exclusively found on this construct. We identified several possible binding sites for Coup-TFII in this fragment: -1269 (A), -901 (B), -837 and -827 (C), -807 (D) and -779 (E).

### Electrophoretic mobility shifts assays

**Figure 5:**
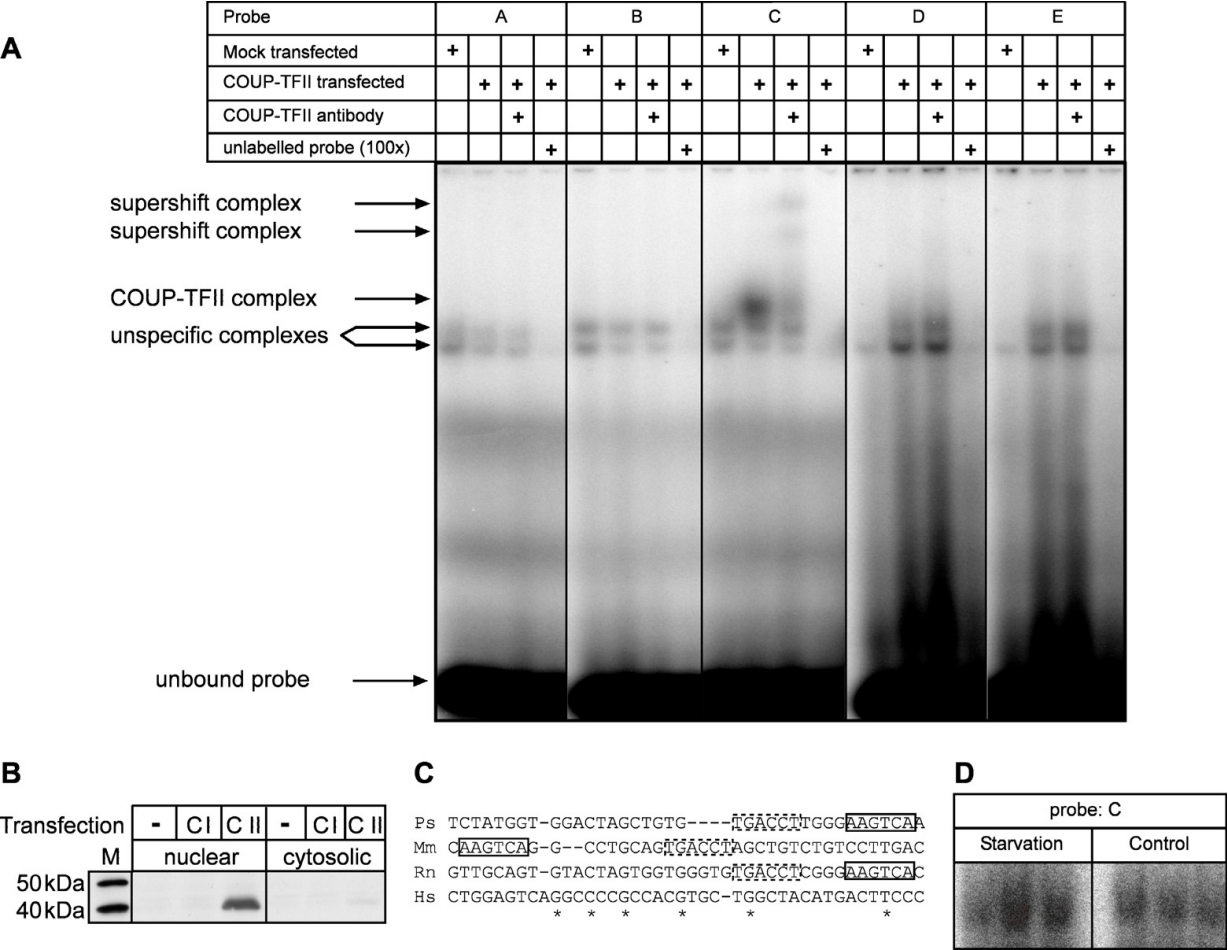
**Coup-TFII binds to a conserved element of the Ucp3 promoter**. (**A**) Electrophoretic mobility shift assay with candidate probes A-E and nuclear extracts of HEK293 cells. Overexpression of Coup-TFII leads to formation of a specific complex on probeC only, that can be supershifted with a Coup-TFII antibody. All complexes are subject to competition with 100 fold molar excess of unlabelled probe C. (**B**) Western Blot of protein fractions of HEK293 cells with a Coup-TFII-antibody. Coup-TFII is specifically detected in the nuclear fraction and absent in untransfected cells. M = Marker, - = mock transfected, CI = Coup-TFI transfected, CII = Coup-TFII transfected. (**C**) Alignment of the human and rodent promoter regions of the *Ucp3 *gene (Ps: -821 to -857). The positions of identical bases are marked (*). The putatively Coup-TFII binding elements (box, dashed box) located on probe C are conserved in all rodent species. In the murine promoter they are found in reverse order slightly more upstream. The human promoter does not feature comparable sequences in the compared region. Rn = *R. norvegicus*, Mm = *M. musculus*, Ps = *P. sungorus*, Hs = *H. sapiens *(**D**) Electrophoretic mobility shift assay with skeletal muscle nuclear extracts from control and food deprived hamsters. The complex shown is of a size comparable to the Coup-TFII complex above. Starvation induces complex formation in comparison to control conditions.

We tested five oligonucleotide probes corresponding to the putative response elements A-E (see above) for binding of Coup-TFII in electrophoretic mobility shift assays. Elements A, B, D and E did not display any specific interaction (Fig. 5A). For element C we were able to show a strong complex formation in the presence of Coup-TFII, that was specifically supershifted by the respective antibody. Binding of proteins to this probe was effectively blocked by competition with the unlabelled oligonucleotide.

The antibody was specific for Coup-TFII and did not bind the close relative Coup-TFI. Virtually all Coup-TFII was detected in the nuclear fraction (Fig. 5B).

Nuclear extracts from skeletal muscle tissue of food deprived (n = 3) and control (n = 3) hamsters were able to form a bandshift on element C of comparable size. In two out of three starvation samples the complex was stronger than in control conditions (Fig. 5D).

The two elements present in oligonucleotide C form a functional repeat as is typical for binding sites of nuclear receptor dimers. This inverse repeat is conserved in the rat *Ucp3 *promoter, and also found slightly more upstream in the murine promoter albeit in reverse order (Fig. 5C).

### Mutational analyses of the Coup-TFII binding element

**Figure 6:**
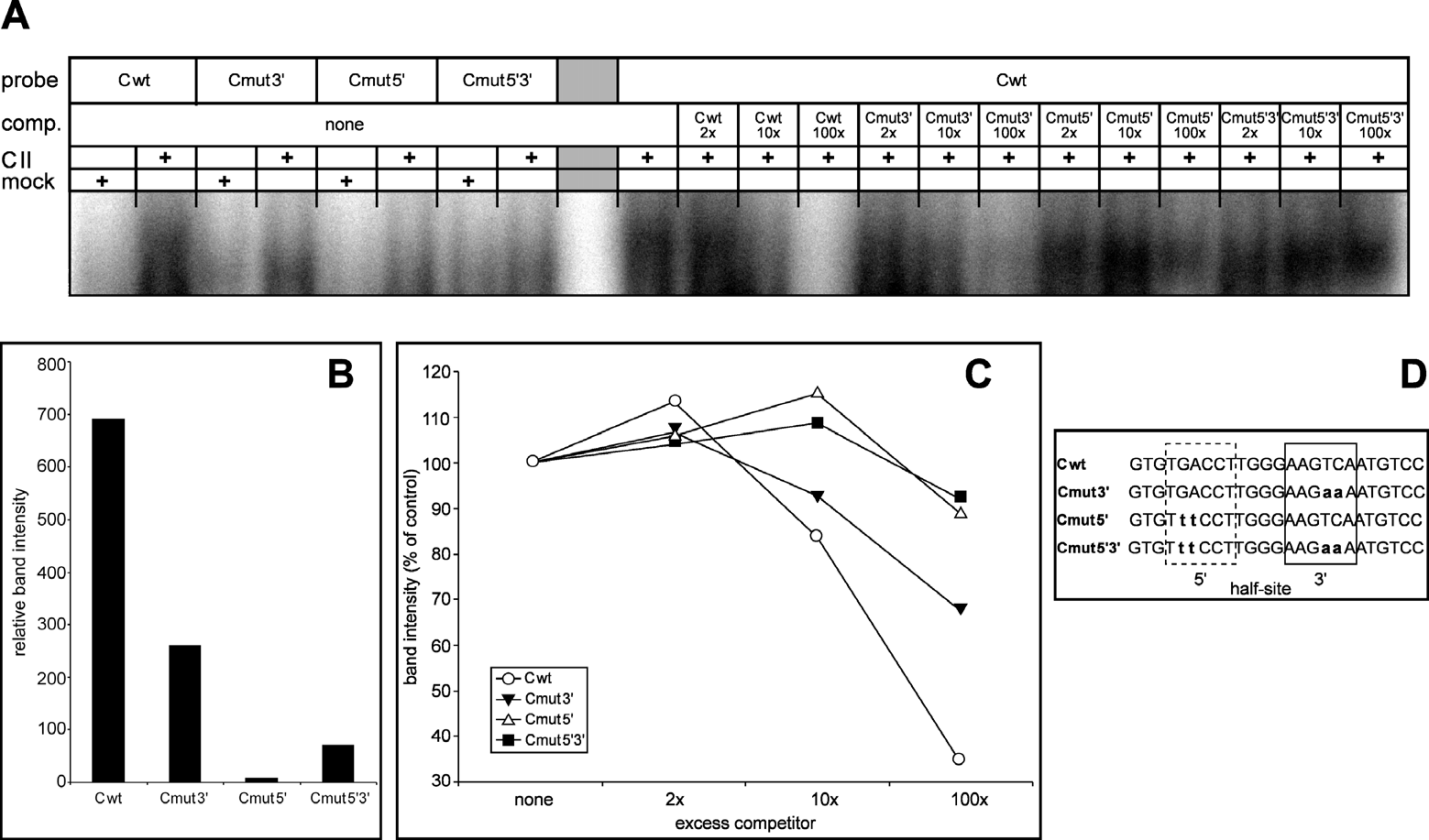
**Disruption of the 5' half-site leads to loss of Coup-TFII binding**. (**A**) Electrophoretic mobility shift assay with element C and mutated derivatives (compare (D)). Bands were quantified, background corrected and analyzed. Probe = labelled oligonucleotide used; comp. = unlabelled competitor oligonucleotide used; CII = nuclear extract of Coup-TFII transfected HEK293 cells; mock = nuclear extract of mock transfected HEK293 cells. (**B**) Quantification of complex intensity on labelled probes Cwt, Cmut5', Cmut3' and Cmut5'3'. Probe Cmut3' retained a lowered ability to bind Coup-TFII while Cmut5'and Cmut5'3'were unable to do so. (**C**) Accordingly unlabelled competitor probe Cmut3' was still able to partially compete for Coup-TFII binding to the labelled wt probe, while Cmut5' and Cmut5'3' were not. (**D**) Overview of the probes used in electrophoretic mobility shift assays. Probe Cwt represents element C, the mutated derivatives are depicted below.

We further analysed the two repeat elements on oligonucleotide C (Cwt) by disrupting the 5' half (Cmut5'), the 3' half (Cmut3') or both (Cmut5'3') on bandshift assay probes (Fig. 6D). We chose to change two bases of the 3' half-site into adenine and two bases of the inverse 5' half-site into thymine correspondingly. These probes were employed either labelled with nuclear extracts of mock or Coup-TFII transfected cells or unlabelled as competitors for the interaction of Coup-TFII with element C (Fig. 6A). All mutated probes displayed a reduced complex formation with Coup-TFII (Fig. 6B). While probes Cmut5' and Cmut5'3' did not show any significant complex formation, probe Cmut3' still formed a complex that was weaker in intensity and slightly smaller than the wt probe. In competition assays the mutated competitor oligonucleotides were less efficient in competing Coup-TFII complex formation on the Cwt probe than the unlabelled Cwt oligonucleotide itself (Fig. 6C). Again Cmut5' and Cmut5'3' generated a similar pattern of no binding of Coup-TFII to these mutated elements. Mutation Cmut3' did not entirely loose its ability to compete complex formation, which is in line with its demonstrated retained capability of interaction with Coup-TFII.

**Figure 7:**
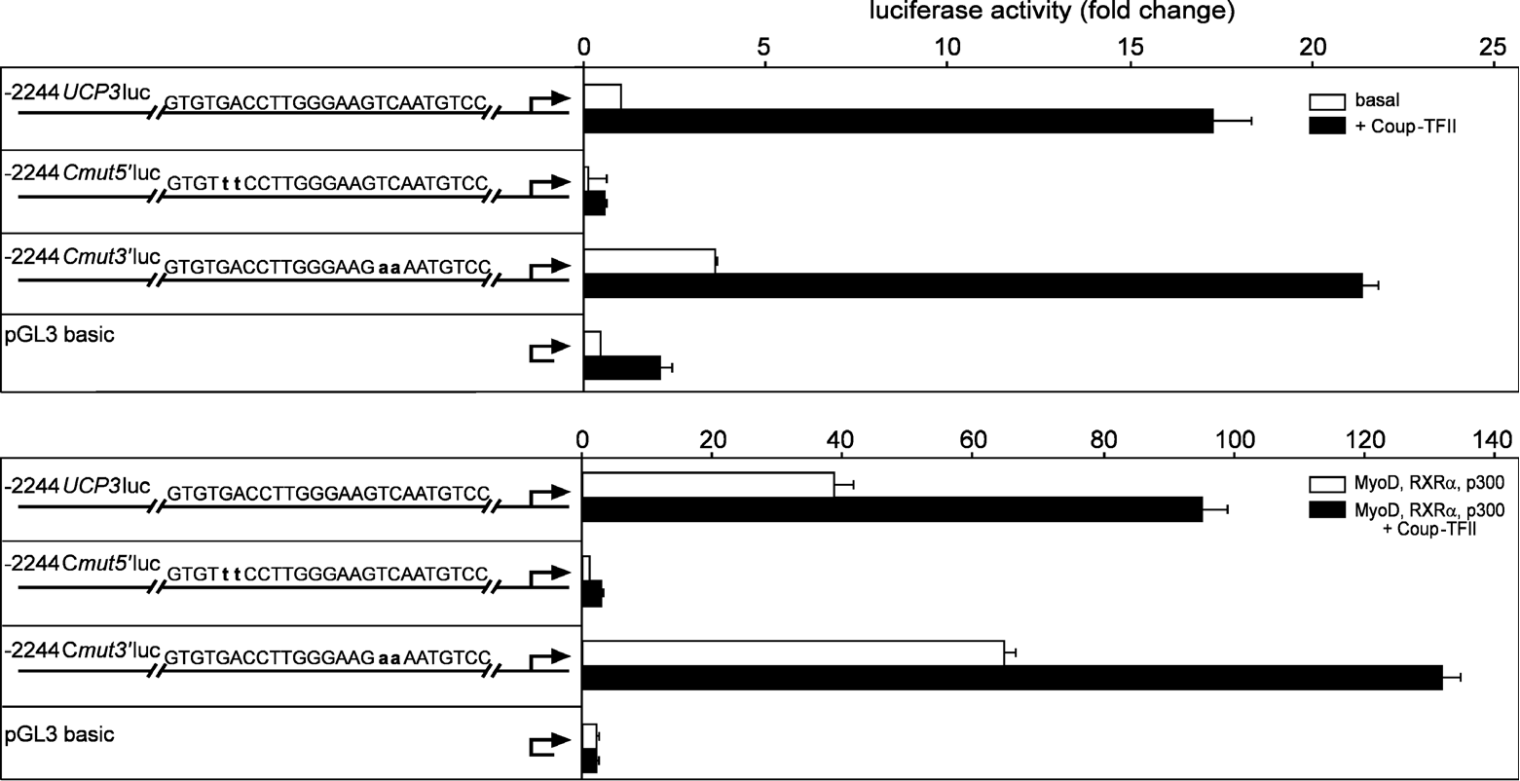
**Disruption of the 5' half-site leads to complete loss of activity**. Reporter gene assays with mutated luciferase constructs. In the upper panel basal luciferase activity and induction by Coup-TFII is shown, in the lower panel the experiment was repeated on a background of MyoD, RXRα and p300. Disruption of the 3' half site on -2244*Cmut3'*luc did not lower activity in any condition tested. Disrupting the 5' half-site on -2244*Cmut5'*luc led to a complete loss of basal activity and responsiveness to Coup-TFII and MyoD, RXRα and p300.

We introduced the same mutations Cmut5' and Cmut3' into our reporter gene vector -2244*UCP3*luc generating -2244*Cmut5'*luc and -2244*Cmut3'*luc (Fig. 7). Disruption of the 3'half-site on -2244*Cmut3'*luc did not lower responsiveness to Coup-TFII alone or on a background of MyoD, RXRα and p300, but did even proportionally increase luciferase activity by a small amount. Conversely, -2244*Cmut5'*luc was devoid of any luciferase activity in all conditions tested and displayed a pattern resembling our empty control vector pGL3 basic.

## Discussion

A prominent hypothesis considers uncoupling protein 3 (Ucp3) to be a crucial component of lipid metabolism with implications for the regulation of body weight and composition [3]. This role is further substantiated by the identification of polymorphisms/alleles in the human *Ucp3 *gene that are associated with an elevated body mass index [28]. A more detailed analysis of the machinery regulating *Ucp3 *transcription is therefore of importance for identifying regulatory networks controlling energy partitioning.

Our comparison of previously characterized *Ucp3 *promoter elements in rat, mouse and human with the hamster sequence, shows full conservation of the binding sites for MyoD, PPARα/RXRα and TR/RXRα heterodimers. The sequence alignment furthermore demonstrates that two TATA-like boxes present in the human promoter [26] are absent in rodent sequences including the hamster. This might prove to be crucial considering, that in the study of Riquet and coworkers (2003) the activities of human constructs were investigated in murine tissue.

The TSS of the human *Ucp3 *gene is quite variable and displays a distinct tissue specificity, whereas in mouse the TSS is located at a single site [29]. Our result of variable TSS in *P. sungorus *demonstrates that the constancy in mouse does not reflect a common trait of rodent species. However, in the hamster the TSS did not show a distinct tissue specificity as found in humans.

By *in silico *analysis we identified Coup-TFII as a candidate transcription factor for the regulation of *Ucp3 *expression. Coup-TFII is a 45 kD nuclear orphan receptor and member of the COUP-TF family. The amino acid sequence of the ligand binding and the DNA binding domain is conserved across species to a very high extent (human vs. Drosophila ~90%) indicating an important role for these domains. Coup-TFII has mainly been described as a crucial factor in developmental processes [21,22].

By qPCR we measured *Ucp3 *and *Coup-TFII *transcript levels in a panel of nine different tissues. We confirmed that BAT and SKM are the major sites of *Ucp3 *mRNA expression. *Coup-TFII *was detected in both tissues, but in line with previous data on human adult tissue distribution [30] is rather ubiquitously expressed with highest levels in kidney, liver and heart. Contrary to reports that *Coup-TFII *is expressed in preadipocytes and myoblasts and downregulated during differentiation [23,31], we were able to detect considerable amounts of *Coup-TFII *transcript in BAT and SKM tissue. Apparantely cell culture systems are devoid of the appropriate physiological stimuli promoting *Coup-TFII *mRNA expression in tissues. This is supported by the absence of Coup-TFII in human embryonal kidney cells as shown in our Western blot experiment (Fig. 5B) despite its presence in human kidney tissue *in vivo *[30]. It has been demonstrated that Coup-TFII plays an important role in the regulation of several genes encoding key metabolic enzymes [18,23-25], which are certainly regulated in terminally differentiated cells.

*Ucp3 *gene expression was upregulated in SKM of food deprived hamsters. In line with the function of Coup-TFII in metabolic regulation we also observed a significant increase of mRNA expression in response to this challenge. Probably owing to the short duration of cold exposure in our study we did not observe a significant cold induced increase in *Ucp3 *mRNA expression in BAT as published previously [5,6]. However, *Coup-TFII *and *Ucp3 *mRNA expression in BAT displayed a similar cold-induced increase in variation. The resemblance of expression levels in SKM and BAT and the response to physiological stimuli culminates in a highly significant correlation of *Coup-TFII *and *Ucp3 *mRNA abundancy under challenged conditions (food deprivation and cold). The absence of such a correlation in the control group suggests that Coup-TFII requires additional factors in order to enhance *Ucp3 *mRNA expression which must be recruited and/or activated beforehand.

We could support this model in reporter gene assays, in which Coup-TFII strongly coactivated *Ucp3 *promotor activity in synergy with PPARα, MyoD, RXRα and/or p300, while the effect of Coup-TFII was much lower alone. These constituents of the well described basal transcription factor complex in our experiments affected *Ucp3 *expression as described previously [15], i.e. strong activation by PPARα/RXRα and p300, dependent on agonist stimulation and presence of MyoD. Coup-TFII specifically enhanced expression in synergy with these factors.

In contrast to our data, Coup-TFII has been shown to negatively interact with MyoD and p300 in SKM, reducing their potential to activate E-box driven reporter gene constructs [17]. This discrepancy may be explained by a differential recruitment of the multiple COUP-TF family cofactors in different physiological environments [e.g. N-Cor, SMRT, RIP140, SRC-1; for a review see [32]]. The specific complex of transcription factors, to which Coup-TFII is recruited, may determine the final function as described for interaction with the glucocorticoid receptor [33]. In general, COUP-TF proteins display a conflicting pattern of positive or negative interaction with nuclear receptors like PPARs or the estrogen receptor depending on the target gene [discussed in [18]]. There are genes where transcription is increased by PPARs and decreased by COUP-TFs [e.g. malic enzyme [19]] as well as genes for which the situation is opposite [e.g. transferrin [20]] or at which both PPARs and COUP-TFs act synergistically [e.g. lipoprotein lipase [18]]. Even a complete reversal of the effect of Coup-TFII on a single target has been reported [23]; transcription of the phosphoenolpyruvate carboxykinase gene is induced or repressed by Coup-TFII in a tissue specific manner.

Interestingly, specific PPARγ agonists upregulate *Coup-TFII *in the heart [24] and also *Ucp3 *gene expression in BAT and SKM [34,35], although to our knowledge no direct interaction of PPARγ with the *Ucp3 *promoter has been verified so far. This effect might therefore be due to PPARγ mediated transactivation of *Coup-TFII*, which in turn enhances *Ucp3 *expression.

We were able to confine the sequence element mediating the Coup-TFII effect to -1307 to -664 by utilizing reporter gene deletion constructs. -2244*UCP3*luc was the only construct being induced by Coup-TFII on a background of MyoD, RXRα and p300 and was exclusively activated high above an unspecific effect by Coup-TFII alone.

Of five candidate elements A-E within this region tested by electrophoretic mobility shift assays we confirmed specific binding of Coup-TFII to element C. Notably, element C did not only show complex formation with overexpressed Coup-TFII but also with nuclear extracts of hamster SKM (Fig. 5D). Extracts of all six animals tested were able to form a complex of comparable size and food deprivation of hamsters led to an increase in band intensity. The element is located at -816 to -840 and constitutes a functional repeat structure. By mutational analyses we were able to confine Coup-TFII binding to the 5' half-site of this element. In reporter gene assays disruption of this site surprisingly led to a complete loss of activity and responsiveness to any treatment tested. While this fact certainly underlines the importance of this element, its indifference to MyoD, RXRα and p300 treatment is in conflict with the retained effect of this treatment on -664*UCP3*luc and -243*UCP3*luc, that are also devoid of this element. It seems that the element exhibits a function beyond direct transactivation by Coup-TFII, that depends on the presence of the surrounding region. These characteristics point towards a model of Coup-TFII deactivating or displacing a so far unknown repressor of *Ucp3 *transcription. This repressor could be expected to bind in proximity to the 5' half-site of elementC, possibly even to the 3' half-site of this same element. In the light of this hypothetical model several formerly negligible seeming facts gain relevancy: Mutated construct -2244*Cmut3'*luc displayed an overall increase of luciferase activity as compared to -2244*UCP3*luc (Fig. 7). Deletion construct -664*UCP3*luc had a higher basal activity and could be activated by MyoD, RXRα and p300 to a higher extent than -2244*UCP3*luc (Fig. 4B). The complex size of bandshift probe Cmut5' was slightly changed (Fig. 6A).

This model alone, however, does not account for the activation of -2244*UCP3*luc by MyoD, RXRα, p300 and PPARα in the absence of cotransfected Coup-TFII. Therefore there would have to be an endogenous Coup-TFII expression in HEK293 cells that we can either not detect with our Western blots or is induced by transfection of factors like MyoD and p300. At present we cannot decide wether repressor displacement/deactivation itself or subsequent direct coactivation is the dominant means of Coup-TFII mediated transcriptional induction.

The conservation of the inverse repeat structure and its Coup-TFII binding site across rodent species suggests an important role for this element; its preservation in the murine promoter despite an obvious event of rearrangement during evolution supports this role. The upstream location of the binding element and its synergy with PPARα resembles a comparable situation, where Coup-TFII directly coactivates the lipoprotein lipase gene in cooperation with PPAR/RXR heterodimers as described previously [18]. Notably lipoprotein lipase is a crucial determinant of fatty acid uptake from circulating triglycerides.

An explanation of the link between increased lipid utilization and the recruitment of Coup-TFII in BAT and SKM is hampered by the lack of knowledge about a specific ligand and the posttranslational mechanism of activation. It is beyond the scope of this study to clarify whether Coup-TFII is directly regulated in response to FFA levels and thus possibly conveys FFA-dependent *Ucp3 *transcription. Our qPCR data indicate that *de novo *synthesis of Coup-TFII is the dominant means of target gene upregulation rather than ligand binding or posttranslational modification. So far, transcription of *Coup-TFII *has been linked to MAP kinase pathway activity [36] and the presence of ETS family transcription factors [37]. Further research is required to clarify whether these mechanisms represent a link between lipid utilization and *Coup-TFII *upregulation.

On the functional level beyond its role in organismic development Coup-TFII has been assumed to be an integral part of the glucose response complex, where it functions as an inhibitor of glucose dependent activators [25]. This hypothesis is based on the ability of Coup-TFII to inhibit upstream stimulatory factor (USF)-dependent transactivation of a glucose response element in the L-type pyruvate kinase gene (*PKLR*) and a similar finding regarding the *ATPA *gene encoding the alpha subunit of the F1F0 ATP synthase complex [38]. Recently, Bardoux and coworkers (2005), after discovering a role for Coup-TFII in insulin secretion and sensitivity, assigned Coup-TFII as an important regulator of glucose homeostasis [39]. Our finding of *Coup-TFII *upregulation in states of augmented lipid oxidation confirms these models and extends them to a new aspect. The inhibition of glucose induced gene transcription may occur in conjunction with a positive regulation of lipid metabolism genes like *Ucp3 *and lipoprotein lipase.

## Conclusion

Coup-TFII is a strong activator of *Ucp3 *gene transcription by binding to an upstream element in the *Ucp3 *promoter as shown by luciferase assays and electrophoretic mobility shift assays. *Coup-TFII *mRNA expression correlates with *Ucp3 *mRNA levels in tissues of hamsters subjected to physiological challenges inducing lipid oxidation. Transcriptional upregulation of the *Coup-TFII *gene in response to these challenges seems to regulate the *Ucp3 *gene in brown adipose and skeletal muscle tissue determining the final appropriate rate of *Ucp3 *mRNA synthesis. The mechanism of Coup-TFII inducing *Ucp3 *transcription seems to involve displacement/deactivation of an unknown repressor. These findings add a crucial component to the complex transcriptional machinery controlling expression of *Ucp3*. At the same time they further manifest a function of Coup-TFII in the regulation of lipid metabolism genes.

## Methods

### Animal experimentation and nucleic acid preparations

Djungarian hamsters (*Phodopussungorus*) were housed under standard laboratory conditions with *ad libitum *access to food (Hamsterzuchtdiät 7014, Altromin) and water unless stated otherwise. All experiments involving animals were conducted in accordance with the German animal welfare law. For tissue dissections hamsters were sacrificed by CO_2 _exposure. Genomic DNA was prepared from spleen with the DNeasy Tissue Kit (Qiagen). We either cold exposed hamsters to 4°C ambient temperature for 24 hours (n = 9) or food deprived hamsters for 48 hours at room temperature (n = 12). A third group of hamsters was kept at room temperature and fed *ad libitum *to serve as control group (n = 12). All hamsters were of an age of 10 to 15 months. We obtained samples of suprasternal BAT and hindlimb SKM (*Musculus quadriceps*) from 9 animals of each group. From three control group hamsters we also dissected samples of inguinal WAT, kidney, liver, heart, lung, spleen and salivary gland. Samples were frozen in liquid nitrogen, total RNA prepared with Trizol (Invitrogen) and quantified photometrically. We dissected the entire leg musculature of three control and starved animals for tissue nuclear protein extracts.

### Primer design, sequencing and sequence analysis

All sequencing reactions were carried out by a commercial service provider (MWG Biotech). Primer sequences were selected using the program Primer3 (Code available at [40]). Primers to amplify fragments of the *Ucp3 *genomic locus were deduced from conserved regions of corresponding genomic sequences from the ENSEMBL mouse and rat genomic databases (ENSMUSG00000032942, ENSRNOG00000017716), from cDNA sequences of the putative 5' adjacent gene [GenBank: AU020772, AI170065] and from *P. sungorus Ucp3 *cDNA [Genbank: AF271265]. PCR products were cloned and sequenced. Assembled contigs were resequenced with homologous primers and a minimum 5-fold coverage. To analyse the *P. sungorus Ucp3 *transcript 2 μg of RNA were reverse transcribed into cDNA with oligo-dT-primers and Superscript II reagents (Invitrogen). We deduced PCR primers from the assembled genomic contig to amplify, clone and sequence two overlapping fragments representing exon1 to exon6 and exon2 to exon7 of the *Ucp3 *transcript, respectively. Sequencing results were visualized, edited and assembled in the GAP4 module of the Staden Sequence Analysis Package [41]. All sequence alignments were conducted with ClustalW [42]. Promoter sequences were analyzed for putative transcription factor binding sites by considering results obtained with the programs AliBaba2.1, MATCH™ and PATCH™ [43] all based upon the TRANSFAC^® ^database [44].

### Mapping the transcriptional start site (TSS)

To determine TSS by 5' rapid amplification of cDNA ends (5' RACE), 1 μg of SKM and BAT total RNA was reverse transcribed into RACE-ready first-strand cDNA using an amplification kit (BD SMART RACE, Clontech). We PCR-amplified the 5'-end with the *Ucp3 *specific reverse primer ATGGCTTGAAATCGGACCTTCACCAC combined with the kit primer mix according to the manual. Products were cloned into pCR2.1-TOPO (Invitrogen) and independent clones were picked and sequenced.

### Real time qPCR

We cloned a fragment of *P. sungorus Coup-TFII *[GenBank: DQ244042] representing parts of exon2 and 3 by deducing heterologous primers from conserved regions of the respective mouse and rat transcripts. Nested, homologous primers were inferred for qPCR analysis. SuperScript^® ^III Platinum^® ^SYBR^® ^Green Two-Step qRT-PCR Kit (Invitrogen) was used to reverse transcribe total RNA prepared as described above and to measure mRNA expression levels on an iCycler IQ (Biorad). The PCR Mix was supplemented with 20 nM Fluorescein (Biorad). Amplification efficiency was calculated based on dilution series standard curves by the Biorad iCycler IQ 3.0 software and used to determine starting quantity levels (PCR base line substracted) normalized to *β-actin *levels. Primers: *β-actin *AGAGGGAAATCGTGCGTGAC, CAATAGTGATGACCTGGCCGT; *Coup-TFII *ATATCCCGGATGAGGGTTTC, AAAGTCCCAGTGTGCTTTGG; *Ucp3 *AGGAAGGAATCAGGGGCTTA, TCCAGCAGCTTCTCCTTGAT. Group differences were tested for significance with the Mann-Whitney rank sum test, correlations were calculated by the Spearman rank order method (SigmaStat 3.1, Systat Software).

### Vector origins and construction

Expression vectors pCMV-SPORT6 harbouring the complete coding sequence of human *Coup-TFII *(IMAGE:5177487), *COUP-TFI *(IMAGE:2824138) and murine *MyoD *in pME18S-FL (IMAGE:1499265) were obtained from the "Deutsches Resourcenzentrum für Genomforschung" (RZPD, Berlin) and verified by sequencing. Human full length *RXRα *in pSG5 was kindly provided by A. Baniahmad (Gießen, Germany), human *p300 *in pCMVβ by C. Platzer (Jena, Germany) and human *PPARα *in pCMV7 by H. Shimano (Tokyo, Japan).

A region comprising a BamHI fragment from -2244 to +38 of the *Ucp3 *gene was amplified by Pfu polymerase (Fermentas). It was cloned into a BglII site of pGL3 basic (Promega) exploiting the endogenous, compatible BamHI sites to generate -2244*Ucp3*luc. Subsequently, three different deletion constructs were cloned (outlined in Fig. 4B). By restriction with BglII and religation we created -2244BglIIdel *Ucp3*luc, bearing a 1161 bp deletion from -1307 to -146. The construct -664*Ucp3*luc was generated by restriction of -2244*Ucp3*luc with the compatible enzymes XbaI and NheI, but owing to a XbaI site in the vector backbone we applied a partial restriction followed by gel purification and religation of the appropriate fragment. We constructed -243*Ucp3*luc by restriction of -664*Ucp3*luc with BfrBI and SmaI and religation of the agarose gel purified larger fragment.

For mutational analyses of element C we constructed two further reporter gene constructs -2244*Cmut5'*luc and -2244*Cmut3'*luc by PCR overlap extension site directed mutagenesis as described previously [45]. To mutate the first half-site (Cmut5') we used the overlapping mutated primers TCAAGGACATTTTCTTCCCA and CCTTGGGAAGAAAATGTCCT, to mutate the second half-site (Cmut3') ACTTCCCAAGGAAACACAGC and GCTGTGTTTCCTTGGGAAGT. For both primer pairs we designed the outer primers TCACTGTTGTCTCTGCTGCC and GCAGCAGCCATCCTTAGAAC to produce an amplicon including the two endogenous BglII sites described above. The two resulting mutated fragments were cloned into pGEM-Teasy (Promega) and sequenced to verify the introduced mutation. BglII-fragments derived from these vectors were subcloned into -2244BglIIdel *Ucp3*luc and again sequenced to guarantee successful subcloning of the respective mutated site.

### Cell culture and luciferase assays

We utilized the human embryonal kidney cell line HEK293 as a heterologous system for our reporter gene studies. Cells were grown in DMEM supplemented with 10% FCS and split every second day. For luciferase assays cells were detached by trypsin and passaged onto 12 well-plates. Cells were transfected by the calcium-phosphate method using the ProFection Kit (Promega). We used 25 ng reporter gene construct, 1.25 ng phRL-tk (Promega), 500 ng for each expression vector and added empty pcDNA3 (Invitrogen) to a final mass of 2 μg and added 100 μl of precipitate solution per well to the medium. For every vector combination we prepared three replicate wells. Cells were incubated with the transfection mixture for 16 hours, medium was changed and cells harvested 24 hours later. When 10 μM of the PPARα ligand Wy14,643 was applied as a stimulant, it was added in DMSO during the medium change. In all measurements without stimulant, medium was supplied with the same volume of DMSO only. Luciferase activity was measured with the Dual Luciferase Reporter Assay System (Promega). Initial experiments revealed that cotransfection of cells with vectors driving expression of nuclear receptors systematically affected phRL-tk activity, a known handicap of this system [46]. We therefore used primary, unnormalized data for activity calculations. To validate these data we measured protein content by the Bradford method and confirmed that this normalization did not result in values significantly different from unnormalized data. All results are shown as fold changes relative to the basal activity of the respective construct used.

### Electrophoretic mobility shift assay

HEK293 cells were harvested in 200 μl homogenisation buffer (10 mM HEPES, 1.5 mM MgCl_2_, 10 mM KCl, 0.5 mM DTT, 20 mM NaF), homogenized in a glass potter and centrifuged (3300 g, 15 min, 4°C). The pellet was resuspended in 100 μl low salt buffer (20 mM HEPES, 1.5 mM MgCl_2_, 0.2 mM EDTA, 0.5 mM DTT, 20 mM NaF, 25% (v/v) glycerol) and subsequently mixed with 100 μl high salt buffer (= low salt buffer with 1.2 M KCl). The mixture was agitated vigorously for 30 min at 4°C and centrifuged (25,000 g, 30 min, 4°C). The supernatant containing nuclear proteins was purified with a Microcon YM-50 column (Millipore), aliquoted and stored at -80°C. All above mentioned buffers were supplied with protease inhibitors (Complete Mini Protease Inhibitor Cocktail Tablet, Roche) and phosphatase inhibitors (Phosphatase Inhibitor Cocktail II, Sigma). Protein concentrations were measured by the Bradford method with BSA as a standard.

For tissue nuclear extracts we excised the complete leg musculature of a hamster and grinded it in liquid nitrogen to yield a fine powder. The powder was treated as described above with the following modifications. We used 1 ml homogenisation buffer, washed the pellet in 1 ml homogenisation buffer and subsequently used 200 μl of high and low salt buffer. We did not employ the concentrator column for tissue extracts.

Complementary oligonucleotides were annealed by heating to 80°C in TE (10 mM Tris/HCl, 1 mM EDTA) and slow cooling to room temperature. Double strands were further purified by elution from a 12% polyacrylamide gel. Three pmol of the resulting doublestrand were endlabelled with T4 polynucleotide kinase and [γ-^32^P]ATP and purified with a ChromaSpin+10-TE column (Clontech). Sequence of oligonucleotide probes (sense strand): (A) GCCCTCCAGTCTGACTCCTCGTAGC, (B) AGCCTCCAATGACTTGTCATGGAG, (C) GTGTGACCTTGGGAAGTCAATGTCC, (D) TGTCCTTGAAGTTCAGTTTTCTGT, (E) AGTAAGCATTGACACATGAGGGTT. Sequence of mutated element C oligonucleotide probes (sense strand): GTGTTTCCTTGGGAAGTCAATGTCC (Cmut5'), GTGTGACCTTGGGAAGAAAATGTCC (Cmut3'), GTGTTTCCTTGGGAAGAAAATGTCC (Cmut5'3').

For gel retardation assays we incubated 3 μg (HEK293) or 20 μg (tissue) nuclear extract with ~7.5 fmol probe for 20 min on ice in a final volume of 2 μl containing 4% (v/v) glycerol, 1 mM MgCl_2_, 0.5 mM EDTA, 0.5 mM DTT, 50 mM NaCl, 10 mM Tris/HCl (pH 7.5) and 50 μg/ml poly(dIdC)·poly(dIdC). Samples were analyzed by electrophoresis in a 5.2% nondenaturing polyacrylamide gel at 4°C and 200 V for 3 hours in 0.5 × TBE. The gel was exposed to a phosphoscreen for 18 hours. For competition assays we included a 2–100 fold molar excess of unlabelled oligonucleotide in the reaction. For supershift assays we added 1 μl of Coup-TFII antibody (Santa Cruz, sc6576-X). The specificity of this antibody and the presence of Coup-TFII protein in the nuclear fraction of transfected cells was confirmed by Western blot.

Densitometrical quantification of band intensities was performed with the software ImageJ 1.34s [47]. All values were background corrected.

## Authors' contributions

TF performed all experiments and computational analyses and drafted the manuscript. KR collaborated in computational analyses and provided knowledge on database handling and bioinformatics. MP supplied technical expertise and participated in experimental design. XL helped in performing experiments shown in Fig. 2 and 3. MK coordinated this study, provided its conceptual basis, participated in experimental design and helped to draft the manuscript. All authors read and approved the final manuscript.
